# Soft Drink Intake in Europe—A Review of Data from Nationally Representative Food Consumption Surveys

**DOI:** 10.3390/nu15061368

**Published:** 2023-03-11

**Authors:** Janette Walton, Anna Wittekind

**Affiliations:** 1 Department of Biological Sciences, Munster Technological University, T12 P928 Cork, Ireland; 2 Independent Nutrition Consultant, Great Missenden HP16 0BS, UK

**Keywords:** soft drinks, sugar-sweetened beverages, SSBs, diet drinks, energy drinks, national dietary surveys, food consumption, free sugars, added sugars

## Abstract

Public health interest in reducing the intake of sugar-sweetened soft drinks has resulted in various guidelines and initiatives related to their consumption, together with an increase in availability and sales of low and no-sugars versions. The aim of this review was to gain insight regarding individual-level amounts and types of soft drinks consumed across the lifecycle as reported in nationally representative surveys in Europe. The review highlighted significant gaps and challenges regarding the availability of recent country-specific soft drink consumption data including heterogeneity in categorisations used in reporting soft drinks. Nonetheless, crude estimates of mean intake (across countries) indicated that total soft drinks and soft drinks with sugars was highest in adolescents and lowest in infants/toddlers and older adults. For infants/toddlers, crude mean intakes of soft drinks with reduced/no sugars were higher than soft drinks with sugars. The review also found that consumption of total soft drinks is decreasing with a shift to consumption of soft drinks with reduced/no sugars in replacement of sugars-containing soft drinks. This review provides valuable insight into what data are currently available on soft drink consumption in Europe with heterogeneity in categorisations, terminology, and definitions of soft drinks observed.

## 1. Introduction

‘Soft drinks’ are an umbrella term for a class of water-based non-alcoholic beverages with no regulatory or standard definition, however the term ‘soft’ serves to distinguish them from ‘hard’ (alcoholic) drinks; and tea, coffee, milk, cocoa, and undiluted vegetable and fruit juices are generally not considered to be soft drinks [1].

There is significant public health interest in the consumption of soft drinks mainly due to their contribution to the intake of overall added/free sugars and associations between the intake of sugar-sweetened beverages (SSBs) and the risk of obesity and non-communicable diseases [2,3], with SSBs broadly considered to be soft drinks with added sugars [4]. Added sugars are defined as all mono- and disaccharides added to foods by the manufacturer, cook, or consumer and free sugars additionally include sugars naturally present in honey, syrups, and fruit juices. Guidelines from the World Health Organisation (WHO) recommend an intake of <10% of energy intake for free sugars for both children and adults and the European Food Safety Authority (EFSA) recommends that the intake of added and free sugars should be as low as possible in the context of a nutritionally adequate diet [2,3].

Furthermore, food-based dietary guidelines (FBDG) across the world generally recommend water and milk as preferred drinks and many recommend limiting the consumption of foods and beverages high in sugar, including SSBs [5,6,7]. For the most part however, FBDG refer to SSBs without any reference to energy/sugars content and mostly without any guidance in relation to the consumption of alternative soft drinks (e.g., those with no or lower levels of sugars) [5,6,7]. Public health organisations and governments have recommended various policy interventions to reduce added/free sugars intake, including interventions to reduce the intake of SSBs and/or their sugars’ content [8,9]. These recommendations have included levies on soft drinks with some countries imposing levies on all soft drinks or only SSBs, with others imposing levies dependent on the sugars content of the beverage [10,11].

Although diet (no sugars) soft drinks have been available from as early as the 1950s, government, and industry initiatives to reduce added/free sugars intake have increased the availability of new or reformulated soft drink products with low or no sugars. For example, the proportion of sales of low/no calorie soft drinks in the European Union (EU) has increased from 2016 to 2021 from 23 to 30%, with a decrease in the proportion of sales of regular soft drinks (not reduced in sugars) (from 77–70%) [12]. Whilst sales data are acknowledged as useful to understand overall market demands, changes to nutrition composition, and year-on-year trends for informing policy [13], understanding and monitoring of individual-level consumption data on food categories (including soft drinks) is necessary to provide information at a demographic level which is imperative for targeted policy-making [14]. Few studies have collated data on the intake of soft drinks across countries either globally/or in Europe and those that have conducted such reviews have still relied mainly on food availability data or food frequency questionnaires (FFQs) and have estimated the intake of SSBs only with no information on their sugars content [15,16]. These studies suggest significant variance in intake of SSBs between countries, regions and age groups and estimate a mean intake of 326 mL/d for children and adolescents in WHO regions with high-dietary related burden of disease [16] and a mean intake of 0.58 8 oz servings/d (137 mL/d) for adults globally with 0.27–0.39 servings (64–92 mL/d) for adults across European regions [15].

The complexity of categorising and quantifying soft drink consumption for dietary assessment and epidemiological studies [4,17] and for informing health policy [18] has been highlighted previously and is now even more important as SSBs can contain a wide range of sugar levels. There is also public health interest in some other specific sub-categories of soft drinks such as ‘energy’ drinks, which are also increasing in market share. These drinks are generally marketed for boosting mental alertness and physical performance and typically contain caffeine and other functional ingredients and are available both with sugars and as reduced/no sugars alternatives [19,20]. In an attempt to harmonise individual-level food consumption data across the EU for exposure assessment, a food classification system (FoodEx2) has been developed. However, not all countries may have classified their most recent data and the current categories for soft drinks may not accurately reflect those captured in the original survey and may not be sufficient to capture the level of sugar content without linking to an updated food composition or brand database [21]. It would therefore be informative to understand what data on soft drink consumption has been captured across national dietary surveys in global regions such as in Europe.

Hence, the aim of the current review is to collate country-specific individual-level data on soft drink consumption in Europe from national dietary surveys to investigate to what extent it can provide insight into recent amounts and types of soft drink consumption and changes in the amounts or types of soft drink consumed over time.

## 2. Materials and Methods

### 2.1. Identification and Extraction of Data on Soft Drink Intake

Data were identified and extracted between March and July 2022. Recent reviews of dietary surveys [22,23,24] were used to identify a list of countries in the WHO European region that have undertaken dietary surveys and the dates that these surveys were conducted.

Inclusion criteria for the current review were that the data were (i) nationally representative of the country (ii) summarised (no raw data were analysed) and (iii) collected at the individual level. As food and beverage intake data derived from national dietary surveys are typically published in reports, rather than peer-reviewed papers, the data presented in this review were primarily collected from various reports and publications from the individual countries. Accordingly, a formal systematic review was not performed. For some countries (Germany and Denmark), where data on soft drinks had been shown in a figure or discussed in a report but without actual numbers, researchers were contacted directly and very kindly provided the data. Peer review publications were used to obtain additional data where available [25]. For some studies, data were translated into English from the native language in which the report was written. The EFSA Comprehensive European Food Consumption Database, which uses the 7-level FoodEx2 Exposure Hierarchy, was used to source chronic intake data for some countries but only if the original survey report was not found by the authors [26]. In cases where the EFSA database was used, drinks typically considered as ‘soft drinks’ were identified as separate sub-categories within ‘water and water-based beverages’ which is at Level 1 in the FoodEx2 Exposure Hierarchy. ‘Soft drinks’ data were extracted at exposure Level 3, with the various sub-categories of ‘Diet’ from Level 6 (‘Diet soft drink with caffeine/fruit juice/flavours’) and data on ‘Drink mixes’ and ‘Functional drinks’ was extracted from Level 3, with the sub-category of ‘Energy drinks’ (considered a ‘Functional drink’) from Level 4′. Drink mixes were all treated as liquid as intakes of powders are minimal and the dilution factor of 7 was used to reconstitute [27]. There was no ‘diet’ element in the hierarchy for functional drinks, drink mixes or energy drinks.

There was no limit to the study size included and data from all direct dietary assessment methodologies were included (food diaries, diet recalls or food-frequency questionnaires). For countries and population groups where there was a previous survey also available, data were extracted to see if it was possible to examine changes in consumption over time.

### 2.2. Recategorisation of Data

To assess the available evidence on soft drink consumption in Europe, published intake data were initially collated using the classification/categorisation in the original reports, prior to a secondary analysis of the intake data using new summary-level categorisations. Data for soft drinks did not account for any potential under-reporting within the self-reported data. Data were compiled according to approximate age groups to align as near as possible with those in the EFSA food consumption database [26] and data are reported on mean intakes per day (in grams rounded to the nearest whole number assuming 1 mL ≡ 1 g) for the total population, with when available, % consumers and mean intakes for consumers only for each category reported in the original source. No formal statistical analysis was performed on the original data sets, and the results are purely descriptive. For each country and age-group, the data used were (to the best of the authors’ knowledge) the most recent that had been published prior to August 2022.

New summary categories were derived based on a nutritional perspective of the categories in the original reports with all soft drink consumption data from the original reports being assigned either directly to one of the new categories or derived (summed/split), depending on how the data were categorised in the original reports. Data were only assigned to a sub-category when the wording was obvious/explicit. Where data were presented by sub-categories of age-group/sex only (and not for total population), the data were weighted to give one value per country for each category and crude estimates of intakes per category are reported as a range, mean and median for descriptive purposes only. It is important to note that there are significant limitations in comparing food intakes across countries due to different country specific classification/categories for soft drinks and due to different time points for data collection.

Where data were available from previous surveys and allowed comparison (i.e., age group and soft drink categorisation matched), the magnitude and direction of change were recorded. Changes in intake exceeding 10% of the earliest intake data were noted as a change.

## 3. Results

### 3.1. Categories of Soft Drinks

The categories of soft drinks reported across countries and even across different surveys within the same country were heterogenous with reported categories including total soft drinks (with different definitions) and some further categorised in various ways including: by energy content, presence of sugars/sweeteners, by carbonation, by functionality and by a mix of these categories. Where countries separated carbonated drinks (soda, fizzy drinks) from cordials/syrups/squash or fruit juice mixes, it was not always clear whether the water for dilution was included in the reporting.

The new categories that we derived from the data focussed on nutritional health (rather than food safety/planetary health) and included ‘total soft drinks’, ‘soft drinks with sugars’ and ‘soft drinks with reduced/no sugars’ (Table 1). Drinks containing less or no sugars were either indicated by naming, which appeared to align at least by name to the EU nutrition claims regulation [28] or by the presence of ‘artificial sweeteners’ or ‘with sweetener’. The wording of the nutrition claim did not always align with the regulations (e.g., ‘diet’), and the sugars or energy level of drinks containing sweeteners was not apparent. Energy drinks were also extracted as a category in this review due to the public health interest surrounding their consumption [19,20].

For ‘total soft drinks’ categorised by the authors, terminology in the original reports included ‘soft drinks’ using a variety of definitions of included drinks, with almost all including carbonated and non-carbonated beverages (with sugars/alternative sweeteners). Some countries however also included other beverages such as sweetened cappuccino, iced teas, ciders and low-alcohol drinks, almond milks, nectars, energy drinks and waters. Other terminology for total soft drinks included ‘Soft drinks, cordials etc., ‘Soda and soft drinks’, ‘Soft drinks (with %fruit less than in nectars)’, ‘Carbonated/soft/isotonic drinks, diluted syrups’, ‘Juice drinks/soda’, ‘Syrups and soft drinks’, ‘Soft and energy drinks’ and ‘Soft drinks, sports and energy drinks’.

For soft drinks categorised by the authors as ‘soft drinks with sugars’, terminology used by individual countries included ‘sugary’, ‘sugar-sweetened’, ‘with added sugar’, ‘with sugar’, ‘not low calorie’, ‘sweet- with added sugar’, ‘non-diet’ and ‘sweetened’ (differentiated as containing sugars by another category in the same survey named ‘artificially sweetened’). For soft drinks categorised by the authors as ‘soft drinks with reduced/no sugars’, terminology used in individual countries included ‘diet’, ‘light’, ‘low calorie’, ‘with sweetener’, ‘energy reduced’, ‘reduced calorie’, ‘without sugar’, ‘artificial sweet’, ‘artificially sweetened’, ‘with artificial sweeteners’, ‘no added sugar’ and ‘sugar free’. Only one country in this review (Iceland) attempted to provide intermediate categories for soft drinks with reduced sugars content (other than diet/no sugars) and they reported by carbohydrate (CHO) content (g) as well as category type (‘carbonated soft drinks with added sugar containing >10 g CHO’, ‘sugary sports drinks that contain 4–6 g CHO’ ‘water drinks that contain 2–6 g CHO’ and ‘sugar free soft drinks’).

### 3.2. Soft Drink Consumption

Data on soft drinks consumption were available (in at least one age group) for 28 countries of the 53 countries in the WHO European region with dietary survey collection dates spanning from 2001 to 2020 across age-groups and countries. Data from the EFSA food consumption database was used for Austria (apart from adults), Croatia, Cyprus, Czech Republic, Estonia, France, Greece, Hungary, Latvia, Romania and Slovenia and Germany (for infants and toddlers only). Countries for which data were available were all from the EU and the European continent i.e., not from countries additionally covered by WHO European region.

#### 3.2.1. Infants/Toddlers (~6 mo–3 y)

For infants /toddlers, soft drink consumption data were available for 15 countries with data on total soft drinks, soft drinks with sugars and soft drinks with reduced/no sugars available for 15, 3 and 4 countries, respectively (Table 2). There were no data available on the intakes of energy drinks in this age-group. The mean daily intake (across countries) of total soft drinks in infants/toddlers ranged from 1 g/d to 389 g/d (mean 60 g/d; median 18 g/d), soft drinks with sugars ranged from 9 g/d to 50 g/d (mean 28 g/d; median 24 g/d) and soft drinks with reduced/no sugars from 2 g/d to 96 g/d (mean 40 g/d; median 31 g/d). 

#### 3.2.2. Children (~4–9 y)

For children, soft drink consumption data were available for 20 countries with data on total soft drinks, soft drinks with sugars and soft drinks with reduced/no sugars available for 18, 9 and 7 countries, respectively (Table 3). The mean daily intake (across countries) of total soft drinks ranged from 18 g/d to 515 g/d (mean:131 g/d; median: 100 g/d), soft drinks with sugars ranged from 34 g/d to 196 g/d (mean: 105 g/d; median: 117 g/d) and soft drinks with reduced/no sugars ranged from 1 g/d to 110 g/d (mean: 44 g/d; median 41 g/d). The mean daily intake of energy drinks was only available for one country (Austria) with a mean daily intake of 1 g/d which was consumed by 1% of the observed population.

#### 3.2.3. Adolescents (~10–17 y)

Soft drink consumption data were available for adolescents in 24 countries with data on total soft drinks, soft drinks with sugars and soft drinks with reduced/no sugars available for 22, 12 and 10 countries, respectively (Table 4). Data on energy drinks were available for nine countries (Table 4). The mean daily intake (across countries) of total soft drinks ranged from 38 g/d to 655 g/d (mean 194 g/d; median 141 g/d), soft drinks with sugars ranged from 57 g/d to 354 g/d (mean 152 g/d; median 142 g/d), soft drinks with reduced/no sugars ranged from 1 g/d to 105 g/d (mean 41 g/d; median 33 g/d) and energy drinks ranged from 0 g/d to 11 g/d (mean 4 g/d; median 2 g/d). 

#### 3.2.4. Adults (~18–64 y)

Soft drink consumption data were available for adults in 28 countries with data on total soft drinks, soft drinks with sugars and soft drinks with reduced/no sugars available for 26,14 and 12 countries, respectively (Table 5). Data on energy drinks were available for eight countries (Table 5). The mean daily intake (across countries) of total soft drinks across countries ranged from 20 g/d to 291 g/d (mean 125 g/d; median 106 g/d), soft drinks with sugars ranged from 42 g/d to 149 g/d (mean 95 g/d; median 91 g/d), soft drinks with reduced/no sugars ranged from 0 g/d to 112 g/d (mean 36 g/d; median 26 g/d) and energy drinks ranged from 0 g/d to 116 g/d (mean 18 g/d; median 2 g/d).

#### 3.2.5. Older Adults (~>65 y)

Soft drink consumption data were available for older adults in 23 countries with data on total soft drinks, soft drinks with sugars and soft drinks with reduced/no sugars available for 22, 9 and 9 countries, respectively (Table 6). Data on energy drinks were available for two countries (Table 6). The mean daily intake (across countries) of total soft drinks ranged from 8 g/d to 113 g/d (mean 43 g/d; median 29 g/d), soft drinks with sugars ranged from 8 g/d to 59 g/d (mean 28 g/d; median 24 g/d), soft drinks with reduced/no sugars ranged from 0 g/d to 17 g/d (mean 7 g/d; median 6 g/d). The mean daily intake of energy drinks was only available for two countries and ranged from 2 g/d to 48 g/d (mean 25 g/d; median 25 g/d).

#### 3.2.6. Patterns of Soft Drink Intake

Based on data as reported and one data point per country, crude estimates of mean intakes of total soft drinks in Europe were 60 g/d for infants/toddlers, 131 g/d for children, 194 g/d for adolescents, 125 g/d for adults and 43 g/d for older adults. For soft drinks with sugars, intakes were 28 g/d for infants/toddlers, 105 g/d for children, 152 g/d for adolescents, 95 g/d for adults and 28 g/d for older adults. For soft drinks with reduced/no sugars, mean intakes were 40 g/d for infants/toddlers, 44 g/d for children, 41 g/d for adolescents, 36 g/d for adults and 7 g/d for older adults. For energy drinks, mean intakes were 1 g/d for children (from very limited data), 4 g/d for adolescents, 18 g/d for adults, and 25 g/d for older adults.

These crude estimates should however be interpreted with caution due to the age of some of the available data together with the significant variances observed in intakes across countries, as can be seen by the large ranges reported in the data above. Nonetheless, these findings may indicate that the mean intake of total soft drinks and soft drinks with sugars was highest in adolescents and lowest in infants/toddlers and older adults. The mean intake of soft drinks with reduced/no sugars was similar across age groups except for older adults (who had a much lower intake). While data were limited in some-age-groups for energy drinks, mean intakes were low overall (1–25 g/d range across age groups,) and intakes were higher in adults and older adults than in children or adolescents. For each age-group except for infants/toddlers, soft drinks with sugars were the highest, then soft drinks with reduced/no sugars followed by energy drinks. For infants/toddlers, soft drinks with reduced/no sugars were higher than those with sugars.

### 3.3. Changes in Soft Drink Intake over Time

Table 7 presents data on the mean intake of soft drinks over time where more than one nationally representative survey was available with comparable data. For infants/toddlers, data were available at two time points for three countries. These data showed a decrease in the intake of any available category of soft drinks except for Norway (for artificial sweet drinks) where there was no notable change. For children, data were available for six countries with overall findings that the intake of soft drinks with sugars has decreased over time in line with an increase/no change to the intake of soft drinks with reduced/no sugars. For adolescents, data were available for six countries showing a decrease in total soft drinks and soft drinks with sugars and an increase in soft drinks with reduced/no sugars. An increase was also noted in the intake of energy drinks in adolescents in one country (Ireland) for which these data were reported. For adults, data were available for seven countries showing an overall decrease in the intake of soft drinks. For older adults, data were available for two countries with a decrease in soft drinks noted for both countries.

## 4. Discussion

### 4.1. Overall Findings

This review has collated individual-level soft drink consumption data across the lifecycle from nationally representative surveys in Europe and has attempted to classify the various soft drink categories used in national surveys from a nutrition perspective to investigate if the data can provide recent estimates of soft drinks intake which are necessary for meaningful policy discussion. The review has highlighted significant gaps and challenges regarding the availability of recent country-specific data relating to soft drink consumption and has also highlighted significant heterogeneity in the categorisations used in national dietary surveys for reporting soft drink consumption in the context of overall food consumption. Based on very crude estimates from the available data with significant variance observed in intakes across countries, mean intakes of total soft drinks and soft drinks with sugars were highest in adolescents and lowest in infants/toddlers and older adults. The mean intake of soft drinks with reduced/no sugars was lower than that of soft drinks with sugars for all age groups except for infants/toddlers. Mean intakes of energy drinks were lower than that of other soft drink categories and were higher in adults and older adults than in adolescents with few/no data available for children. The review also found that the consumption of total soft drinks appears to be decreasing and there is an apparent shift to the consumption of soft drinks with reduced/no sugars in replacement of sugar-containing soft drinks in some age categories where comparisons were possible.

### 4.2. Challenges in Data Availability and Quality

The importance of having recent, high-quality, country-specific, nationally representative food consumption data to inform nutrition policy has been well acknowledged in the literature; together with the associated gaps and challenges surrounding data availability and use [14,22,23,84]. This review has focused specifically on soft drink consumption and has highlighted challenges relating to both the availability and standardisation of data pertaining to their consumption. Of the 53 countries in the WHO European region, data on soft drink consumption (of any type) and for at least one age group were available from only 28 countries with any available data being from EU countries and the European continent leaving significant gaps in the data for some parts of the region. While the most recent survey for each country was extracted (by population group), some surveys dated back 18–20 years perhaps questioning the reliability of these data in reflecting current estimates. Furthermore, dietary surveys across countries varied greatly regarding dietary assessment methodologies utilised, age groups included, statistics reported, and categorisation of food groupings. This review found that for soft drink consumption (all types), there were most data available for adults (28 countries) and the least available data for infants and toddlers (15 countries). Most recent surveys employed 24 h dietary recalls for adult populations while food records were more common in infants/toddlers and children. This is in keeping with EFSA guidelines for the harmonisation of food consumption data in the EU for dietary exposure assessment [85].

The variance in definitions used to reflect categories of soft drinks consumption has previously been acknowledged in the literature together with the associated challenges for epidemiological studies and for informing policy [4,18] and similar findings was observed in this review, not only across countries but also across different surveys within the same country. The term ‘soft drinks’ was used frequently to reflect total soft drink consumption but with a variety of definitions for included drinks, depending on reporting country. This variance was also observed for sub-categories of soft drinks including those with sugars and with reduced/no sugars with countries categorising soft drinks by energy content, presence of sugars/sweeteners, by carbonation, by functionality and by a mix of these categories.

Notwithstanding the challenges surrounding these definitions and categorisations, this review identified significant gaps in available data in all categories including categories of importance from a nutrition perspective. Only a few countries (approx. half in each population group) had available data on the consumption of reduced/no sugars soft drinks and even less on the consumption of energy drinks (in certain age groups). More data on these latter categories together with standardisation of terminology will be important for informing nutrition policy related to soft drink consumption [19,86]. With respect to the move to a reduction in sugar content in soft drinks, new categories may be needed going forward for classifying all soft drinks, including cordials/syrups/squashes and energy drinks with different levels of sugars. The EU food classification system (FoodEx2) may be able to support standardisation of terminology (for EU countries at least) however the current categorisation only includes a diet option for soft drinks (not drink mixes or functional drinks) and may not be able to capture new formulations of soft drinks with varied sugars content) [18]. An option may be to align categories and terminology with those already defined by nutrition claims such as low sugars, sugars free, with no added sugars or reduced sugars which would reflect the fact that soft drinks can contain sugars or sweeteners or both in combination to achieve a range of sugars and calorie levels [28].

### 4.3. Soft Drink Consumption (Including Intakes and Changes over Time)

A key finding of this study was that it does not seem possible to reliably estimate recent soft drink intakes across European countries due to differences in categorisations, age ranges, dates of surveys, and methodologies employed. However, it may be possible to estimate recent intakes within a country and population group, but its value may still be limited by the granularity of the data collected (i.e., purely soft drinks or sub-categorisations). Similarly, it may be possible to identify trends when the categorisations and age groups have remained stable within a country over sequential surveys. However, again its value may be limited by the granularity of the data collected.

Hence, the crude estimates that are reported here should be interpreted with caution and with an understanding of these limitations; and an observation of patterns of intake across categories or population groups may be more valuable than any attempt to quantify intakes. In this regard, our crude estimates of mean intakes of soft drink consumption in Europe suggest that intakes of soft drinks with sugars were higher than soft drinks with reduced/no sugars in all population groups (except for infants/toddlers) which aligns with sales data on these proportions [12,87] and mean intakes of energy drinks were low/negligible at the population level. In keeping with other studies, we found that soft drink consumption and soft drinks with sugars were highest in adolescents and adults and lowest in older adults and infants/toddlers [2,88].

To add to the very limited data available on quantitative estimates of soft drinks, this review showed (very crudely) that for total soft drinks, mean intake across countries is less than one 8 oz serving a day (194 g) for adolescents and approx. half a serving for children (131 g) and adults (125 g) and one-quarter to one fifth of a serving per day (43–60 g) for older adults and infants/toddlers, respectively. A recent systematic review of SSB consumption in children and teenagers (2–18 y) included countries from the following WHO regions with high dietary-related burden of disease, Western Pacific, Southeast Asia, and the Americas (using mainly FFQs) and found a considerable variation of intake across countries from 115 mL (Australia) to 710 mL (China) with a pooled synthesis of 326 mL. Our estimates of 105 g/d for children and 152 g/d for teenagers for soft drinks with sugars are in keeping with the lower range of these findings. For adults, a review in 2010 of global, regional and national consumption of beverages (including SSBs) estimated a mean intake of SSBs of 137 mL/d for adults globally and approx. 64–92 mL/d for adults across European regions which is broadly in keeping with our estimate for adults of 95 g/d of soft drinks with sugars (albeit at the lower end). EFSA have previously reported consumption of energy drinks for European countries and whilst the data (reported for consumers only, in volumes per month) are not directly comparable to ours, they also showed higher intakes in adults and adolescents compared with children [89].

Findings from the current review, albeit for a very limited range of countries, did suggest an overall decrease in the consumption of soft drinks together with a shift to the consumption of soft drinks with reduced/no sugars in replacement of sugar-containing soft drinks in some age-categories. This finding is in keeping with sales data from the EU of an increase of low/no calorie soft drinks in line with a reduction of regular soft drinks (not reduced in sugars) as a proportion of total sales from 2016–2021 [12] and also reflects sales data reported for the US and Australia [87,90]. These trends may be in part due to policy interventions and/or industry initiatives however lack of data on motivations of specific food choice within dietary survey reports does not allow us to comment with any certainty on this. With recently renewed guidelines on reducing sugar intake, it will be important to continue to monitor changes in SSBs and reduced/no sugars soft drinks as well as that of total soft drinks consumption [86]. Interestingly, despite the renewed emphasis in guidelines on sugars, FBDG generally only refer to reduction of SSBs and many are silent regarding other categories of soft drinks with lower levels of or no sugars [5,6,7].

### 4.4. Strengths and Limitations

Strengths of the present review include the large number of studies included and the use of the most recent data for each country including the reporting of all soft drinks data as published in the original reports. The attempt to categorise soft drink consumption from the available data by nutritionally relevant categories is also a strength. The review is limited by gaps in the available data and some nuance may have been lost when translating soft drink categories from reports written in the native language. Another limitation that may be important in the context of estimating soft drinks consumption is that some aspects of representativeness of the data could not be considered such as seasonal data, reporting on weekday/weekend days and sociodemographic of the population groups but it is hoped that the design of the individual surveys will have accounted for this as much as possible.

## 5. Conclusions

This review provides a valuable insight into what data are currently available on soft drink consumption, (including subcategories of nutritional importance) in European populations, in particular the heterogeneity in categorisations, terminology, definitions, and types of soft drinks included. If the limitations are addressed, the data can provide a broad evidence base for future monitoring, which may be useful for informing nutrition practice, research, and policy. However, some findings from this review, in particular crude mean intakes, must be interpreted with caution due to vast gaps in recent available data for some countries and gaps and heterogeneity in reporting of subcategories of soft drinks. More regular and ongoing monitoring of country-specific food consumption data is necessary to address questions on both nutrition and food safety and for a complete picture, it will be important to extend the data to European countries not included in the presented work.

## Figures and Tables

**Table 1:** Soft drink categories derived from the categories reported in National Food Consumption Surveys in the WHO European Region.

Country (Year)	Age-Group	Total Soft Drinks	Soft Drinks with Sugars	Soft Drinks with Reduced/No Sugars	Energy Drinks	Other *
Andorra (2017–18) [29]	12–75 y	**Soft drinks**				
Austria (2017) [30]	18–64 y	**Soft drinks**				
Belgium (2014–15) [31]	3–64 y		**Sugary drinks** (Including ‘lemonades’ (all soft drinks, sports drinks and energy drinks) and flavoured water with energy content > 5 kcal/100 g; and cappuccino)			
Belgium (2004) [32]	60–74 y, 75 y+		**Soft drinks** (lemonades)(distinguished as with sugar by another category-soft drinks (lemonades) light)	**Soft drinks (lemonades), light**		
Bulgaria (2014) [33,34]	1–74 y; 75 y+	**Soft drinks** (energy drinks reported separately for 19–74 y)			**Energy drinks**	
Denmark (2011–13) [35]	4–75 y		**Cordial** (distinguished as with sugar by another category Juice, cordial light)	**Juice, cordial, light**		**Ice tea, Cider** (Low/no alcohol sweetened drink)
			**Fizzy soft drinks, sugar-sweetened**	**Fizzy soft drinks, light**		
Estonia (2014) [25]	18–74 y	**Soft drinks, cordials, etc.** (also includes energy drinks, near water, nectars, and fruit drinks)				
Finland (2017) [36]	18–74 y		**Juice drinks** (sugar sweetened) (Juice drink diluted with water from juice concentrate)	**Drinks with artificial sweeteners** (Artificially/partially artificially sweetened juice or soft drink)		**Sports drinks, recovery drinks**
			**Soft drinks sweetened with sugar** (Soft drink, cola beverage, energy drink) flavoured water			
Germany (2014–17) [37]	3–17 y		**Sugary soft drinks including energy drinks** (Soft drinks include lemonades, fizzy drinks, fruit spritzers and fruit juice drinks, further drinks such as malt beer, ice teas and energy drinks)	**Reduced calorie soft drinks**		
Germany (2005–06) [38]	14–80 y	**Soft drinks, total**	**Sugary soft drinks**	**Energy-reduced soft drinks**		
Iceland (2007) [39]	3 y, 5 y	**Sweet fruit drinks** and **soft drinks** (reported separately)				
Iceland (2001–12) [40] (2003–04) [41]	6 y 9 y,15 y	**Soda and soft drinks** (Includes carbonated beverages (with sugar/artificial sweeteners) and sugary fruit/berry drinks. Excludes carbonated water—with/without flavourings) (split further into soft drinks and soda for 6 y olds)				
Iceland (2010–11) [42]	18–80 y	**Soda and soft drinks** (Sugary and sugar-free soft drinks—and soft drinks, including sports drinks and water drinks containing at least 2 g CHO/100 g)	**Sugary soft drinks** (Carbonated soft drinks with added sugar, containing> 10 g CHO)	**Sugar-free soft drinks** Carbonated sugar-free soft drinks with sweeteners		**Fruit drinks** Sugary and sugar-free fruit and berry drinks
				**Sports drinks** Sugary sports drinks that contain 4–6 g CHO		
				**Water drinks** Water drinks that contain 2–6 g CHO		
Ireland (2010–2011) [43]	1–4 y		**Soft drinks not low calorie**	**Soft drinks low calorie**		
Ireland (2017–2018) [44] (2019–20) [45]	5–12 y 13–17 y		**Soft drinks with added sugar**	**Soft drink with no added sugar**	**Energy Drinks** (13–17 y only)	
Ireland (2008–10) [46]	18 y+		**Non-diet carbonated soft drinks**	**Diet carbonated soft drinks**		**Squash, cordial and FJ drinks**
Italy (2005–06) [47]	0.1–64 y 65 y+	**Soft drinks** (all carbonate beverages (e.g., cola, soda, ginger ale, orange, tonic water) with sugar /sugar-free, ice-tea, ice herbal tea, energy drinks, sport drinks, syrups to be diluted (inc. almond milk). Not inc. juice drinks)				
Latvia (2018–20) [25]	19–64 y	**Soft drinks, cordials, etc.**				
Lithuania (2013–14) [48]	19–75 y	**Soft drinks (with % fruit less than in nectars)**				
Netherlands (2012–16) [49]	1–18 y; 19–79 y	**Carbonated/soft/isotonic drinks, diluted syrups**				
Norway (2020) [50]	6 mo–17 y		**Total sugary drinks** (Includes soda with sugar, juice with sugar and nectar)			
			**Juice drink, sweetened**	**Juice drink, artificially sweetened**		
			**Soda, sweetened**	**Soda, artificially sweetened**		
Norway (2020) [50]	12 mo and 2 y		**Sweet drinks (with added sugar)** Includes Nectar, juice with sugar, soda with sugar (reported separately as well so nectar can be removed)	**Artificial sweet drinks** (Includes Juice without sugar, soda without sugar)		**Nectars**
			**Juice with sugar**	**Juice without sugar**		
			**Soda with sugar**	**Soda without sugar**		
Norway (2015) [51]	9 y, 13 y		**Sweet drinks with added sugar** (Juice, nectar, soda with added sugar)	**Light juice drinks/soda**		
Norway (2010–11) [52]	18–70 y	**Juice drinks/soda**	**Juice drinks/soda etc. with sugar**	**Light juice drinks/soda**		**Juice drink/soda unspecified type**
Portugal (2015–16) [53]	3–64 y; 65–84 y	**Soft drinks** (Carbonated and non-carbonated soft drinks, lemonade, tonic water, energy drinks, and juice concentrates). (100% juices, isotonic drinks and alcohol-free beer and cocktails are excluded)				**Nectars** (Fruit and/or vegetables nectars and light nectars)
Spain (2012–14) [54] (2014–15) [55]	6 mo–17 y;18–74 y	**Soft drinks**				
Sweden (2003) [56]	4–9 y	**Syrups and soft drinks**	**Syrups**	**Syrups, light**		
			**Soft drinks**	**Soft drinks, light**		
Sweden (2016–17) [57]	11–18 y	**Soft and energy drinks**	**Soft drink with sugar**	**Soft drink with sweetener**	**Energy drink**	
Sweden (2010–11) [58]	18–80 y	**Soft drinks, sports and energy drinks**	**Soft drinks, sports and energy drinks with sugar**	**Soft drinks, sports and energy drinks with sweetener**		
Switzerland (2014–15) [59]	18–75 y	**Soft drinks** (Sweetened and sugar-free soft drinks, sports and energy drinks, fizzy drinks, ice tea, diluted syrup, drinks made with fruit juices (e.g., lemonades, nectars), and alcoholic drink substitutes (e.g., alcohol-free beers), ‘schorle’ (i.e., juices mixed with water))				
UK (2011) [60]	4–18 mo		**Squash/soft drink (not low-calorie)**	**Squash/soft drink (low-calorie)**		
UK 2016/2017–2018/2019 [61]	1.5–18 y; 19–64 y; 65 y+		**Sugar-sweetened soft drink (not low-calorie)**			
EFSA Categories [21]		**Soft drinks** (not including functional drinks or drink mixes)		**Diet soft drinks**	**Energy drinks**	**Functional drinks and drink mixes**

Bold denotes categorisations as per the original report, with any additional information not in bold. * included in total soft drinks category but not further categorised; mo: months; y: years.

**Table 2:** Soft drink intake in infants and toddlers (<4 years) in the WHO European Region, as reported in National Dietary Surveys and by new categorisation.

Country	Method	Year of Survey	Age Range (y)	N	Soft Drink Category	Mean (SD) (g/d)	% Consumers	Mean (SD) Consumers (g/d)	Soft Drink Intake by New Category (g/d)			
									Total	With Sugars	Reduced/No Sugars	Energy Drinks
Bulgaria [33]	2 × 24 h recall	2014	1–2	35	Soft drink	4 (17)						
Cyprus [62] *	3/4 day food record	2014	1–2	275	Soft drinks	0 (1)	1	8 (2)	1			
					Drink mixes	0 (3)	4	10 (9)				
Estonia [63] *	Food record, 24-h recall	2014	≤11 mo	504	Soft drinks	0 (4)	0	56 (63)	11			
			1–2	268		31 (69)	32	97 (92)				
France [64] *	3 × 24-h food record	2014–2015	1–2	139	Soft drink	30 (82)	29	106 (126)	37			
					Drink mixes	1 (2)	8	7 (5)				
Germany * [26]	Food record	2001	≤11 mo	159	Soft drink	19 (51)	26	74 (78)	104			
		2006	1–2	348	Soft drinks	143(214)	72					
					Functional soft drinks	0 (5)	2	69 (20)				
Hungary [65] *	2 × 24 h food record and recall	2018–2020	1–2	535	Soft drinks	15 (71)	8	192 (182)	29		2	
					*of which diet*	2						
					Drink mixes	2 (9)	8	22 (24)				
Iceland [39]	3-day food record	2007	3	225	Sweet fruit drinks	48 (87)			61			
					Soft drinks	13 (34)						
Ireland [43]	4-day food record	2010–2011	1	126	Soft drinks not low calorie	21 (66)	21	96 (115)	146	50	96	
					Soft drinks low calorie	68 (144)	29	233 (180)				
			2	124	Soft drinks not low calorie	49 (103)	42	117 (131)				
					Soft drinks low calorie	103 (181)	45	228 (210)				
			3	126	Soft drinks not low calorie	52 (91)	47	111 (106)				
					Soft drinks low calorie	104 (156)	52	198 (167)				
			4	124	Soft drinks not low calorie	77 (181)	53	145 (228)				
					Soft drinks low calorie	111 (177)	50	223 (196)				
Italy [47]	3-day food record	2005–2006	0.1–2.9	52	Soft drinks	2 (10)	3 (10)	4 (10)	2			
Latvia [66] *	2 × 24 h recall and food record	2013	≤11 mo	171	Soft drinks	5 (25)	5	93 (66)	7			
			1–2	242		8 (29)	10	83 (51)				
Netherlands [49]	2 × 24 h recall	2012–2016	1–3	672	Carbonated/soft/isotonic drinks, diluted syrups	389	83^a^	468 ^a^	389			
Norway [50]	Semi-quantitative FFQ	2020	6 mo	2182	Total sugary drinks	0 (2)	1	18 (15)	18	9	9	
					*Juice drink, sweetened*	0 (1)	0.2	27 (24)				
					*Soda, sweetened*	0	0	0				
					Juice drink, artificially sweetened	0	0.5	17 (17)				
					Soda, artificially sweetened	0	0	0				
			12 mo	1957	Sweet drinks (with added sugar)	5 (31)	11					
					*Nectar*	1 (7)	5					
					*Juice with sugar*	5 (31)	7					
					*Soda with sugar*	1 (22)	1					
					Artificial sweet drinks	2 (15)	7					
					*Juice without sugar*	2 (15)	6					
					*Soda without sugar*	0 (1)	1					
			2	1413	Sweet drinks (with added sugar)	40 (94)	48					
					*Nectar*	11 (42)	19					
					*Juice with sugar*	26 (75)	36					
					*Soda with sugar*	3 (12)	9					
					Artificial sweet drinks	32 (101)	31					
					*Juice without sugar*	29 (99)	27					
					*Soda without sugar*	3 (11)	10					
Slovenia [67] *	2 × 24 h recall	2017	≤11 mo	294	Soft drinks	0.1 (2)	1	20 (25)	11			
			1–2	3343		12 (56)	12	101 (131)				
Spain [54]	2 × 1-day food record	2012–2014	6–12 mo	289	Soft drinks	0			2			
			1–3	326	Soft drinks	2	NR	NR				
					Isotonic drink	1 (12)	0.3	200 (141)				
UK [60,61]	4-day food record	2011	4–6 mo	329	Squash/soft drink (not low-calorie)	3 (14)	6	43	77	24	53	
					Squash/soft drink (low-calorie)	4 (26)	7	57				
			7–9 mo	630	Squash/soft drink (not low-calorie)	5 (24)	9	55				
					Squash/soft drink (low-calorie)	18 (72)	18	102				
			10–11 mo	449	Squash/soft drink (not low-calorie)	15 (65)	15	104				
					Squash/soft drink (low-calorie)	43 (123)	26	165				
			12–18 mo	1275	Squash/soft drink (not low-calorie)	42 (119)	26	158				
					Squash/soft drink (low-calorie)	87 (169)	46	189				
		2016/2017–2018/2019	1.5–3 y	250	Sugar-sweetened soft drink (not low-calorie)	19 (62)						

* denotes data obtained from EFSA Food Consumption database; mo: months; M: male, F: female, ^a^: values represent %consumption days and mean for consumption days only; text in italic represents sub-categories of preceding category.

**Table 3:** Soft drink intake in children (~4–10 y) in the WHO European Region, as reported in National Dietary Surveys and by new categorisation.

Country	Method	Year of Survey	Age Range (y)	N	Soft Drink Category	Mean (SD) (g/d)	% Consumers	Mean (SD) Consumers (g/d)	Soft Drink Intake by New Category (g/d)			
									Total	With Sugars	Reduced/No Sugars	Energy Drinks
Austria [68] *	Food record	2012	3–9	128	Soft drinks	97 (144)	56	172 (154)	103	96	1	1
					*of which diet*	1						
					Functional drinks	6 (37)	3	188 (105)				
					*of which energy*	1 (7)	1	83 (0)				
Belgium [31]	2 × 24 h recall	2014–2015	3–5	454	Sugary drinks	84	44			117		
			6–9	538		145	51					
Bulgaria [33]	2 × 24 h recall	2014	3–6	272	Soft drinks	8 (32)			19			
			7–9	167		37 (87)						
Cyprus [62] *	3/4 d record	2014	3–9	297	Soft drinks	11 (40)	11	105 (72)	18			
					Drink mixes	1 (5)	4	16 (23)				
Czech Rep [69] *	2 × 24 h recall	2003–2004	3–9	389	Soft drinks	137 (210)	51	269 (227)	193			
					Drink mixes	8 (16)	43	18 (20)				
Denmark [35]	7-day record	2011–2013	4–9 (M)	216	Cordial	51			199	118	75	
					Fizzy soft drinks, sugar-sweetened	69						
					Cider	0						
					Ice tea	10						
					Juice, cordial, light	42						
					Fizzy soft drinks, light	27						
			4–9 (F)	205	Cordial	57						
					Fizzy soft drinks, sugar-sweetened	57						
					Juice, cordial, light	55						
					Fizzy soft drinks, light	26						
					Cider	1						
					Ice tea	4						
Estonia [63] *	Food record, 24-h recall	2014	3–9	765	Soft drinks	56 (114)	40	139 (145)	56			
France [64] *	3 × 24-h record	2014–2015	3–9	852	Soft drinks	83 (134)	56	148 (150)	97			
					Drink mixes	2 (9)	17	14 (16)				
Germany [37]	FFQ	2014–2017	3–6 (M)	1742	Sugary soft drinks including energy drinks	101			177	134	41	
					Reduced calorie soft drinks	23						
			3–6 (F)	1634	Sugary soft drinks including energy drinks	88						
					Reduced calorie soft drinks	19						
			7–10 (M)	1772	Sugary soft drinks including energy drinks	211						
					Reduced calorie soft drinks	70						
			7–10 (F)	1683	Sugary soft drinks including energy drinks	131						
					Reduced calorie soft drinks	51						
Hungary [65] *	2 × 24 h record and recall	2018–2020	3–9	537	Soft drinks	46 (117)	21	218 (167)	81	34	12	
					*of which diet*	12						
					Drink mixes	5 (18)	16	30 (36)				
Iceland [39,40,41]	3-day record	2007	5	231	Sweet fruit drinks	56 (82)			179			
					Soft drinks	32 (56)						
	3-day record	2011–2012	6	162	Soda and soft drinks	117 (129)						
					*Soft drinks*	71 (99)						
					*Soda*	46 (76)						
	2 × 24 h recall	2003–2004	9	183	Soda and soft drinks	349 (301)						
Ireland [44]	4-day food record	2017–2018	5–12	600	Soft drinks with added sugar	50 (93)	40	124 (111)	160	50	110	
					Soft drink with no added sugar	110 (201)	47	235 (240)				
Italy [47]	3-day record	2005–2006	3–9.9	193	Soft drinks	28 (53)	32	87 (59)	28			
Latvia [66] *	2 × 24 h recall and records	2013	3–9	782	Soft drinks	24 (63)	19	126 (91)	24			
Netherlands [49]	2 × 24 h recall	2012–2016	4–8	520	Carbonated/soft/isotonic drinks, diluted syrups	515	91 ^a^	567 ^a^	515			
Norway [51]	4-day record	2015	9	636	Sweet drinks with added sugar	145 (149)			195	145	50	
					Light juice drinks/soda	50 (93)						
Portugal [53]	2-day record	2015–2016	3–9	1327	Soft drinks	56	7	248	56			
					Nectars	20	1	203				
Spain [54]	2 × 1-day record	2012–2014	4–9	556	Soft drinks	25 (86)	10	253 (127)	29			
					Isotonic drinks	4 (36)	2	270 (87)				
Sweden [56]	4-day record	2003	4	590	Syrups and soft drinks	187 (149)	94		220	196	20	
					*Syrups*	105 (122)	76					
					*Soft drinks*	63 (76)	61					
					*Syrups, light*	12 (42)	13					
					*Soft drinks, light*	3 (17)	5					
			8–9	889	Syrups and soft drinks	242 (178)	92					
					*Syrups*	104 (124)	66					
					*Soft drinks*	110 (113)	70					
					*Syrups, light*	15 (57)	12					
					*Soft drinks, light*	8 (34)	7					
UK [61]	4-day record	2016/2017–2018/2019	4–10		Sugar-sweetened soft drink (not low-calorie)	52 (111)				52		

* denotes data obtained from EFSA Food Consumption database, M: male, F: female, ^a^: values represent %consumption days and mean for consumption days only, text in italic shows sub-categories of preceding category.

**Table 4:** Soft drink intake in adolescents (~11–17 y) in the WHO European Region, as reported in National Dietary Surveys and by new categorisation.

Country	Method	Year of Survey	Age Range (y)	N	Soft Drink Category	Mean (SD) (g/d)	% Consumers	Mean (SD) Consumers (g/d)	Soft Drink Intake by New Category (g/d)			
									Total	With Sugars	Reduced/No Sugars	Energy Drinks
Andorra [29]	24 h recall + 2 × 24 h records	2017–2018	12–24	155	Soft drinks	112 (225)			112			
Austria [70] *	2 × 24 h recall	2018	10–17	574	Soft drinks	146 (263)	40	363 (305)	156	142	4	8
					*of which diet*	4						
					Functional drinks	10 (48)	5	201 (95)				
					*of which energy*	8 (43)	4	192 (93)				
Belgium [31]	2 × 24 h recall	2014–2015	10–13	449	Sugary drinks	200 (95% CI 191–220)	60			221		
			14–17	479		241 (227–265)	67					
Bulgaria [33]	2 × 24 h recall	2014	10–13	187	Soft drinks	71 (142)			90			
			14–18	338		101 (187)						
Cyprus [71] *	2 × 24 h recall	2014	10–17	271	Soft drinks	45 (95)	28	163 (117)	53			0
					Drink mixes	1 (9)	6	24 (29)				
					Functional drinks	1 (11)	1	90 (74)				
					*of which energy*	0 (5)	0	83 (0)				
Czech Rep [69] *	2 × 24 h recall	2003–2004	10–17	298	Soft drinks	236 (322)	65	364 (336)	292			
					Drink mixes	8 (14)	39	20 (17)				
Denmark [35]	7-day record	2011–2013	10–17 (M)	251	Cordial	70			321	218	87	
					Fizzy soft drinks, sugar-sweetened	186						
					Cider	6						
					Ice tea	13						
					Juice, cordial, light	39						
					Fizzy soft drinks, light	58						
			10–17 (F)	258	Cordial	58						
					Fizzy soft drinks, sugar-sweetened	104						
					Cider	13						
					Ice tea	18						
					Juice, cordial, light	31						
					Fizzy soft drinks, light	47						
Estonia [72] *	24-h recall	2014	10–17	300	Soft drinks	86 (151)	45	191 (175)	86	81	5	
					*of which diet*	5						
France [64] *	3 × 24 h record/recall	2014–2015	10–17	1130	Soft drink	127 (172)	62	207 (178)	142			1
					Drink mixes	2 (7)	10	17 (15)				
					Functional drinks	1 (16)	1	174 (116)				
					*of which energy*	1 (12)	1	143 (92)				
Germany [37]	FFQ	2014–2017	11–13 (M)	1389	Sugary soft drinks including energy drinks	333			459	354	105	
					Reduced calorie soft drinks	103						
			11–13 (F)	1435	Sugary soft drinks including energy drinks	303						
					Reduced calorie soft drinks	109						
			14–17 (M)	1536	Sugary soft drinks including energy drinks	478						
					Reduced calorie soft drinks	125						
			14–17 (F)	1787	Sugary soft drinks including energy drinks	304						
					Reduced calorie soft drinks	86						
Greece [73] *	2 × 24-h recall	2010–2012	10–17	274	Soft drinks	60 (113)	30	200 (123)	62	57	3	2
					*of which diet*	3						
					Functional drinks	2 (16)	1	146 (39)				
					*of which energy*	2 (16)	1	146 (39)				
Hungary [74] *	2 × 24 h recall	2018–2020	10–17	528	Soft drinks	109 (257)	33	334 (357)	139	68	41	2
					*of which diet*	41						
					Drink mixes	4 (21)	12	38 (49)				
					Functional drinks	2 (18)	2	128 (45)				
					*of which energy*	2 (18)	2	128 (45)				
Iceland [41]	2 × 24 h recall	2003–2004	15	183	Soda and soft drinks	553 (491)			553			
Ireland [45]	4-d record	2019–2020	13–18	428	Soft drinks—with added sugar	84 (152)	45	187 (180)	164	84	69	11
					Soft drinks—with no added sugar	69 (146)	31	224 (185)				
					Energy drink	11 (54)	7	169 (137)				
Italy [47]	3-day record	2005–2006	10–17.9 (M)	108	Soft drinks	98 (160)	48	203 (177)	73			
			10–17.9 (F)	139		54 (91)	41	132 (100)				
Latvia [66] *	2 × 24 h recall + records	2014	10–17	620	Soft drinks	38 (94)	22	175 (130)	38			
Netherlands [49]	2 × 24 h recall	2012–2016	9–13	519	Carbonated/soft/isotonic drinks, diluted syrups	642	90 ^a^	712 ^a^	655			
			14–18	524		667	85 ^a^	784 ^a^				
Norway [51]	4-day record	2015	13	687	Sweet drinks with added sugar	204 (258)			276	204	72	
					Light juice drinks/soda	72 (142)						
Portugal [53]	2 × 24 h recall	2015–2016	10–17	632	Soft drinks	161	39	371	161			
					Nectars	38	5	264				
Romania [75] *	2 × 24 h recall	2019	10–17	356	Soft drinks	67 (161)	25	269 (221)	67			0
					Functional drinks	0 (7)	0	125 (0)				
					*of which energy*	0 (7)	0	125 (0)				
Slovenia [67] *	2 × 24 h recall	2017–2018	10–17	484	Soft drinks	71 (153)	32	225 (199)	75	70	1	3
					*of which diet*	1						
					Functional drinks	3 (24)	2	176 (78)				
					*of which energy*	3 (24)	1	187 (77)				
					Drink mixes	0.1 (3)	0.4	31 (42)				
Spain [54]	2 × 24 h recall	2012–2014	10–17	609	Soft drink (total)	67	NR	NR	76			
					Isotonic drink	9 (68)	3	324 (245)				
Sweden [57]	2 × 24 h recall	2016–2017	11–18 (M)	1389	Soft and energy drinks	267 (349)	67		223	188	25	10
					Soft drink with sugar	229 (326)	61					
					Soft drink with sweetener	26 (103)	10					
					Energy drink	12 (59)	5					
			11–18 (F)	1710	Soft and energy drinks	187 (239)	62					
					Soft drink with sugar	154 (210)	55					
					Soft drink with sweetener	24 (87)	11					
					Energy drink	9 (47)	5					
UK [61]	4-day record	2016/2017–2018/2019	11–18	542	Sugar-sweetened soft drink (not low calorie)	142 (199)				142		

* denotes data obtained from EFSA Food Consumption database, M: male, F: female; ^a^: values represent %consumption days and mean for consumption days only, text in italic shows sub-categories of preceding category.

**Table 5:** Soft drink intake in adults(~18–64 y) in the WHO European Region, as reported in National Dietary Surveys and by new categorisation.

Country	Method	Year of Survey	Age Range (y)	N	Soft Drink Category	Mean (SD) (g/d)	% Consumers	Mean (SD) Consumers (g/d)	Soft Drink Intake by New Category (g/d)			
									Total	With Sugars	Reduced/No Sugars	Energy Drinks
Andorra [29]	24 h recall + 2 × 24 h records	2017–2018	12–75	1002	Soft drinks	73 (193)			73			
Austria [30]	2 × 24-h recall	2017	18–64 (M)	736	Soft drinks	248 (401)			167			
			18–64 (F)	1282		121 (262)						
Belgium [31]	2 × 24 h recall	2014–2015	18–39	620	Sugary drinks	209 (192–235)	54			149		
			40–64	606		88 (76–103)	29					
Bulgaria [34]	2 × 24 h recall	2014	19–29	480	Soft drinks	97 (213)			173			116
					Energy drinks	110 (104)						
			30–59	1536	Soft drinks	58 (153)						
					Energy drinks	131 (109)						
			60–74	733	Soft drinks	31 (106)						
					Energy drinks	88 (107)						
Croatia [76] *	3 × 24 h recall	2011–2012	18–64	2000	Soft drinks	95 (201)	37	255 (259)	96	94	1	1
					*of which diet*	1						
					Functional drinks	1 (9)	0	126 (58)				
					*of which energy*	1 (9)	0	126 (58)				
Cyprus [71] *	2 × 24 h recall	2014	18–64	272	Soft drinks	62 (116)	32	198 (127)	91			14
					Drink mixes	2 (7)	6	24 (13)				
					Functional drinks	15 (145)	4	405 (676)				
					*of which energy*	14 (145)	3	480 (745)				
Czech Rep [69] *	2 × 24 h recall	2003–2004	18–64	1666	Soft drinks	109 (231)	30	358 (293)	144			
					Drink mixes	5 (21)	16	31 (43)				
Denmark [35]	7-day record	2011–2013	18–75 (M)	1464	Cordial	45			221	116	91	
					Fizzy soft drinks, sugar-sweetened	110						
					Juice, cordial, light	28						
					Fizzy soft drinks, light	66						
					Cider	4						
					Ice tea	10						
			18–75 (F)	1552	Cordial	27						
					Fizzy soft drinks, sugar-sweetened	52						
					Juice, cordial, light	27						
					Fizzy soft drinks, light	61						
					Cider	10						
					Ice tea	5						
Estonia [25]	24-h recall	2014	18–74 (M)	907	Soft drinks, cordials, etc.	82 (159)			54			
			18–74 (F)	1806		40 (137)						
Finland [36]	2 × 24 h recall	2017	18–74 (M)	780	Juice drinks (sugar sweetened)	60	25	239	119	88	31	
					Soft drinks sweetened with sugar	57	19	305				
					Drinks with artificial sweeteners	38	13	303				
					Sports drinks, recovery drinks	c	4	c				
			18–74 (F)	875	Juice drinks (sugar sweetened)	37	22	167				
					Soft drinks sweetened with sugar	25	12	202				
					Drinks with artificial sweeteners	24	11	211				
					Sports drinks, recovery drinks	c	4	c				
France [64] *	3 × 24 h recall	2014–2015	18–64	1773	Soft drink	86 (186)	37	231 (244)	94			1
					Drink mixes	1 (5)	7	14 (14)				
					Functional drinks	1 (19)	1	164 (126)				
					of which energy	1 (12)	1	155 (88)				
Germany [38]	Diet history interview, food records, 24-h recall	2005–6 NVS II	14–80 (M)	7093	Soft drinks, total	224 (6.05 SE)			151	126	25	
					*Sugary soft drinks*	193 (5.66)						
					*Energy-reduced soft drinks*	31 (2.32)						
			14–80 (F)	8278	Soft drinks, total	88 (3.06)						
					*Sugary soft drinks*	68 (2.68)						
					*Energy-reduced soft drinks*	20 (1.51)						
Greece [73] *	2 × 24-h recall	2010–2012	18–64	260	Soft drink	57 (127)	25	228 (160)	59	55	2	2
					*of which diet*	2						
					Functional drinks	2 (16)	1	145 (36)				
					*of which energy*	2 (16)	1	145 (36)				
					Drink mixes	0 (2)	0	39 (0)				
Hungary [74] *	2 × 24 h recall	2018–2020	18–64	529	Soft drinks	106 (244)	29	366 (331)	143	73	33	9
					*of which diet*	33						
					Drink mixes	4 (19)	9	43 (52)				
					Functional drinks	9 (51)	4	228 (125)				
					*of which energy*	9 (51)	4	228 (125)				
Iceland [42]	2 × 24-h recall	2010–2011	18–80 (M)	632	Soda and soft drinks	274 (358)			238	128	83	
					*Sugary soft drinks*	169 (288)						
					*Sugar-free soft drinks*	66 (211)						
					*Water drinks*	6 (37)						
					*Fruit drinks*	29 (101)						
					*Sports drinks*	4 (32)						
			18–80 (F)	680	Soda and soft drinks	205 (317)						
					*Sugary soft drinks*	89 (199)						
					*Sugar-free soft drinks*	82 (245)						
					*Water drinks*	11 (55)						
					*Fruit drinks*	22 (76)						
					*Sports drinks*	1 (23)						
Ireland [46]	4-day record	2008–2010	18–64	1274	Non-diet carbonated soft drinks	82 (159)	37	223 (193)	118	82	24	
					Diet carbonated soft drinks	24 (79)	14	176 (142)				
					Squash, cordial and FJ drinks	12 (41)	16	74 (77)				
Italy [47]	3-day record	2005–2006	18–64 (M)	1068	Soft drinks	35 (92)	24	147 (138)	29			
			18–64 (F)	1245		24 (62)	21	114 (90)				
Latvia [25]	2 × 24 h recall	2018–2020	19–64 (M)	470	Soft drinks, cordials, etc.	128			92			
			19–64 (F)	541		61						
Lithuania [48]	1 × 24 h recall	2013–2014	19–75	2513	Soft drinks (with % fruit less than in nectars)	20 (107 SE)			20			
Netherlands [49]	2 × 24 h recall	2012–2016	19–79	2078	Carbonated/soft/isotonic drinks, diluted syrups	291	50 ^a^	581 ^a^	291			
Norway [52]	2 × 24 h recall	2010–2011	18–70	1787	Juice drinks/soda	240 (365)			240	125	112	
			18–70 (M)	862	Juice drinks/soda	282 (396)						
					*with sugar*	167 (301)						
					*Light juice drinks/soda*	111 (277)						
					*Juice drink/soda unspecified type*	4 (34)						
			18–70 (F)	925	Juice drinks/soda	202 (329)						
					*with sugar*	86 (207)						
					*Light juice drinks/soda*	113 (268)						
					*Juice drink/soda unspecified type*	2 (19)						
Portugal [53]	2 × 24 h recall	2015–2016	18–64	3102	Soft drinks	100	16	342	100			
					Nectars	21	1	257				
Romania [75] *	2 × 24 h recall	2019	18–64	740	Soft drinks	63 (192)	19	335 (325)	63	63	0	0
					*of which diet*	0.1 (3.7)	0.1					
					Functional drinks	0 (8)	0	145 (28)				
					*of which energy*	0 (8)	0	145 (28)				
Slovenia [67] *	2 × 24 h recall	2017–2018	18–64	385	Soft drinks	43 (127)	19	223 (206)	45	42	1	2
					*of which diet*	1						
					Functional drinks	2 (21)	1	227 (93)				
					*of which energy*	1 (20)	1	265 (92)				
Spain [55]	2 × 24 h recall	2014–2015	18–74	933	Soft drinks	66 (163)	20	338 (210)	69			
					Isotonic drinks	3 (37)	1	328 (161)				
Sweden [58]	4-day record	2010–2011	18–80	1797	Soft drinks, sports and energy drinks	112 (196)	51		112	84	27	
					*Soft drinks, sports and energy drinks with sugar*	84 (148)	44					
					*Soft drinks, sports and energy drinks with sweetener*	27 (123)	15					
Switzerland [59]	2 × 24 h diet history	2014–2015	18–75	2019	Soft drinks	241			241			
UK [61]	4-day record	2016/2017–2018/2019	19–64	1082	Sugar-sweetened soft drinks (not low calorie)	106 (248)				106		

* denotes data obtained from EFSA Food Consumption database, M: male, F: female; ^a^: values represent %consumption days and mean for consumption days only; text in italic shows sub-categories of preceding category; c—denotes data not provided when <30 consumers.

**Table 6:** Soft drink intake in older adults(~>65 y) in the WHO European Region, as reported in National Dietary Surveys and by new categorisation.

Country	Method	Year of Survey	Age Range (y)	N	Soft Drink Category	Mean (SD) (g/d)	% Consumers	Mean (SD) Consumers (g/d)	Soft Drink Intake by New Category (g/d)			
									Total	With Sugars	Reduced/No Sugars	Energy Drinks
Austria [68] *	24-h recall	2010–2012	65–74	67	Soft drinks	34 (133)	13	254 (287)	30	24	5	2
					*of which diet*	5						
					Functional drinks	2 (15)	2	125 (0)				
					*of which energy*	2 (15)	2	125 (0)				
			75+	25	Soft drinks	15 (55)	8	188 (88)				
					*of which diet*	5						
Belgium [32]	2 × 24 h recall	2004	60–74	786	Soft drinks	59 (106)			49	33	16	
					*Soft drinks light*	20 (70)						
			75+	694	Soft drinks	37 (75)						
					*Soft drinks light*	11 (41)						
Bulgaria [34]	2 × 24 h recall	2014	75+	228	Soft drinks	25 (76)			73			48
					Energy drinks	48 (68)						
Cyprus [71] *	2 × 24 h recall	2014	65–74	260	Soft drinks	31 (76)	18	175 (87)	38			
					Drink mixes	1 (6)	7	18 (14)				
Estonia [72] *	24-h recall	2014	65–74	525	Soft drinks	11 (48)	11	103 (111)	12			
					Functional drinks	1 (28)	0.4	358 (400)				
Finland [36]	2 × 24 h recall	2017	65–74 (M)	204	Sugared soft drinks and juices	65			76	59	17	
					Artificially sweetened beverages	13						
			65–74 (F)	247	Sugared soft drinks and juices	54						
					Artificially sweetened beverages	21						
France [64] *	3 × 24 h recall	2014–2015	65–74	384	Soft drinks	26 (107)	15	178 (229)	29			
					Drink mixes	1 (7)	8	18 (17)				
			75+	118	Soft drinks	13 (48)	13	101 (97)				
					Drink mixes	0.2 (2)	2	12 (8)				
Germany [38]	Diet history interview, food record, 24-h recall	2005–2006 NVS II	65–80 (M)	1234	Soft drinks, total	41 (4.43 SE)			31	24	7	
					*Sugary soft drinks*	31 (3.79)						
					*Energy-reduced soft drinks*	10 (2.39)						
			65–80 (F)	1705	Soft drinks, total	24 (3.05)						
					*Sugary soft drinks*	19 (2.57)						
					*Energy-reduced soft drinks*	5 (1.37)						
Greece [73] *	2 × 24-h recall	2010–2012	65–74	257	Soft drinks	27 (114)	10	277 (260)	27	26	1	
					*of which diet*	1						
Hungary [74,77] *	2 × 24 h recall Food record	2018–2020	65–74	527	Soft drinks	35 (151)	11	326 (342)	49		6	
					*of which diet*	6						
					Drink mixes	2 (14)	4	43 (62)				
		2003	75+	80	Soft drinks	46 (107)	28	167 (150)				
					Drink mixes	0.2 (2)	1	17 (0)				
Ireland [46]	4-day record	2008–2010	65+ (M)	106	Non-diet carbonated soft drinks	11 (38)	10	103 (65)	19	8	8	
					Diet carbonated soft drinks	12 (99)	3	419 (505)				
					Squash, cordial and FJ drinks	3 (16)	5	56 (58)				
			65+ (F)	120	Non-diet carbonated soft drinks	6 (22)	8	71 (38)				
					Diet carbonated soft drinks	4 (30)	2	218 (116)				
					Squash, cordial and FJ drinks	3 (13)	6	44 (38)				
Italy [47]	3-day record	2005–2006	65+ (M)	202	Soft drinks	6 (25)	7.4	83 (50)	8			
			65+ (F)	316		9 (41)	6.3	134 (103)				
Latvia [66] *	2 × 24 h recall + diaries	2014	65–74	300	Soft drinks	26 (98)	12	221 (195)	27			
					Drink mixes	0.1 (0.3)	0.3	6 (0)				
Lithuania [48]	1 × 24 h recall	2013–2014	65–75	300	Soft drinks (with %fruit less than in nectars)	9 (46 SE)			9			
Netherlands [49]	2 × 24 h recall	2012–2016	71–79	517	Carbonated/soft/isotonic drinks, diluted syrups	98	30 ^a^	321 ^a^	98			
Norway [52]	2 × 24 h recall	2010–2011	60–70 (M)	217	Juice drinks/soda	132 (245)			113			
			60–70 (F)	164		88 (188)						
Portugal [53]	2 × 24 h recall	2015–2016	65–84	750	Soft drinks	26	0	273	26			
					Nectars	8	0	230				
Romania [75] *	2 × 24 h recall	2019	65–74	356	Soft drinks	20 (101)	8	240 (261)	20	20	0	
					*of which diet*	0.1						
Slovenia [67] *	2 × 24 h recall	2017–2018	65–74	450	Soft drinks	23 (114)	9	243 (294)	23	23	0	
					*of which diet*	0.4						
Spain [55]	2 × 24 h recall	2014–2015	65–74	314	Soft drinks	24 (80)	12	202 (135)	28			
					Isotonic drinks	4 (5)	1	425 (0)				
Sweden [58]	4-day record	2010–2011	65–80	367	Soft drinks, sports and energy drinks	50 (96)			50			
Switzerland [59]	2 × 24 h diet history	2014–2015	65–75	336	Soft drinks	106			106			
UK [61]	4-day record	2016/2017–2018/2019	65+	335	Sugar-sweetened soft drinks (not low calorie)	34 (90)				34		

* denotes data obtained from EFSA Food Consumption database, M: male, F: female ^a^: values represent %consumption days and mean for consumption days only, text in italic shows sub-categories of preceding category.

**Table 7:** Change in soft drink intake over time in the WHO European Region as reported in National Dietary Surveys.

Age Group	Country	Age Range	Previous Survey Year	Most Recent Survey Year	Soft Drink Category	Mean (g/d) Previous	Mean (g/d) Most Recent	% Change	Direction of Change
Infants and toddlers	Bulgaria [33]	1–2	1998	2014	Soft drink	NR	4	NR	↓ *
	Norway [50,78,79]	12 mo	2006–2007	2020	Sweet drinks (with added sugar)	14	5	64	↓
					Artificial sweet drinks	9	2	78	↓
		2	2007	2020	Sweet drinks (with added sugar)	60	40	33	↓
					Artificial sweet drinks	29	32	−10	~=
	UK [61,80]	1.5–3 y	2014/2015–2015/2016	2016/2017–2018/2019	Soft drink (not low-calorie)	37	19	49	↓
Children	Bulgaria [33]	3–6	1998	2014	Soft drink	NR	8	NR	↓ *
		7–9				NR	37	NR	↓ *
	Denmark [35]	4–14	2003–2006	2011–2013	Sugar-sweetened soft drinks	NR	NR	NR	↓ *
	Germany [37]	3–6 M	2003–2006	2014–2017	Soft drinks including energy drinks	175	101	42	↓
		3–6 F				154	88	43	↓
		3–6 M			Reduced calorie soft drinks	20	23	−15	~=
		3–6 F				28	19	32	↓
		7–10 M			Soft drinks including energy drinks	301	211	30	↓
		7–10 F				255	131	49	↓
		7–10 M			Reduced calorie soft drinks	57	70	−23	↑
		7–10 F				29	51	−76	↑
	Ireland [46,81]	5–12	2003–2004	2017–2018	Soft drinks with added sugar	252	50	80	↓
					Soft drinks with no added sugar	78	110	−41	↑
	Norway [51,82]	9 M	2000	2015	Sweet drinks (with added sugar)	323	145	55	↓
		9 F				302	145	52	↓
		9 M			Artificial sweet drinks	47	46	2	~=
		9 F				44	55	−25	↑
	UK [61,80]	4–10	2014/2015–2015/2016	2016/2017–2018/2019	Soft drinks (not low-calorie)	83	52	37	↓
Adolescents	Bulgaria [33]	10–13	1998	2014	Soft drink	NR	71	NR	↓*
		14–18				NR	101	NR	↓ *
	Germany [37]	11–13 M	2003–2006	2014–2017	Soft drinks including energy drinks	439	333	24	↓
		11–13 F				312	303	3	~=
		11–13 M			Reduced calorie soft drinks	54	103	−91	↑
		11–13 F				77	109	−42	↑
		14–17 M			Soft drinks including energy drinks	677	478	29	↓
		14–17 F				413	304	26	↓
		14–17 M			Reduced calorie soft drinks	74	125	−69	↑
		14–17 F				77	86	−12	↑
	Ireland [45]	13–18	2005–2006	2019–2020	Soft drinks with added sugar	213	83	61	↓
					Soft drinks with no added sugar	25	56	−124	↑
					Energy drinks	1	11	−1000	↑
	Norway [51,82]	13 M	2000	2015	Sweet drinks (with added sugar)	499	231	54	↓
		13 F				363	179	51	↓
		13 M			Artificial sweet drinks	55	83	−51	↑
		13 F				54	62	−15	↑
	Sweden [56,57]	11–12 (M)	2003	2016–2017	Soft drinks containing sugars	213	178	16	↓
		11–12 (F)				208	147	29	↓
	UK [61,80]	11–18	2014/2015–2015/2016	2016/2017–2018/2019	Soft drinks (not low-calorie)	191	142	26	↓
Adults	Andorra [29]	15–75	2005	2017–2018	Soft drinks	160	73	54	↓
	Belgium [31,32]	15–64	2004	2014	Sugary drinks	177	150	15	↓
	Bulgaria [34]	19–29	1998	2014	Soft drink	NR	97	NR	↓ *
		30–59				NR	58	NR	↓ *
		60–74				NR	31	NR	↓ *
	Denmark [35]	15–75	2003–2006	2011–2013	Sugar-sweetened sodas	NR	NR	NR	↓ *
	Iceland [42,83]	18–80	2002	2010–2011	Soft drinks, total	261	238	9	↓
	Ireland [46,81]	18–64	1997–1999	2008–2010	Non-diet carbonated	86	82	5	~=
					Diet carbonated	35	24	31	↓
					Other (squashes, cordials and fruit juice drinks)	20	12	40	↓
	UK [61,80]	19–64	2014/2015–2015/2016	2016/2017–2018/2019	Soft drinks (not low-calorie)	129	106	18	↓
Older adults	Bulgaria [34]	75+	1998	2014	Soft drink	NR	25		↓ *
	UK [61,80]	65+	2014/2015–2015/2016	2016/2017–2018/2019	Soft drinks (not low-calorie)	40	34	15	↓

* Recent reports report a decrease in this category. ~= denotes approximately equal to.

## Data Availability

Data is contained within the article.
